# Augmented Reality in Dentistry: Enhancing Precision in Clinical Procedures—A Systematic Review

**DOI:** 10.3390/clinpract14060178

**Published:** 2024-10-28

**Authors:** Francesco Puleio, Vincenzo Tosco, Rosario Pirri, Michele Simeone, Riccardo Monterubbianesi, Giorgio Lo Giudice, Roberto Lo Giudice

**Affiliations:** 1 Department of Biomedical and Dental Sciences and Morphofunctional Imaging, Messina University, 98100 Messina, Italy; francesco.puleio@unime.it; 2 Department of Clinical Sciences and Stomatology (DISCO), Università Politecnica delle Marche, 60126 Ancona, Italy; v.tosco@pm.univpm.it (V.T.); r.monterubbianesi@univpm.it (R.M.); 3 Independent Researcher, 98124 Messina, Italy; rosariopirri97@gmail.com; 4 Department of Neuroscience, Reproductive Science and Dentistry, University of Naples Federico II, 80138 Naples, Italy; michele.simeone@unina.it; 5 Department of Human Pathology of Adults and Developmental Age, University of Messina, 98100 Messina, Italy; roberto.logiudice@unime.it

**Keywords:** augmented reality, AR, virtual reality, VR, precision medicine, computer-assisted surgical procedure, head-mounted displays, HMD

## Abstract

**Background:** Augmented reality (AR) enhances sensory perception by adding extra information, improving anatomical localization and simplifying treatment views. In dentistry, digital planning on bidimensional screens lacks real-time feedback, leading to potential errors. However, it is not clear if AR can improve the clinical treatment precision. The aim of this research is to evaluate if the use of AR-based instruments could improve dental procedure precision. **Methods:** This review covered studies from January 2018 to June 2023, focusing on AR in dentistry. The PICO question was “Does AR increase the precision of dental interventions compared to non-AR techniques?”. The systematic review was carried out on electronic databases, including Ovid MEDLINE, PubMed, and the Web of Science, with the following inclusion criteria: studies comparing the variation in the precision of interventions carried out with AR instruments and non-AR techniques. **Results:** Thirteen studies were included. **Conclusions:** The results of this systematic review demonstrate that AR enhances the precision of various dental procedures. The authors advise clinicians to use AR-based tools in order to improve the precision of their therapies.

## 1. Introduction

The digitalization that has impacted the dental field in the last decade has brought significant improvements both in clinical workflow and patient comfort. Furthermore, the introduction of augmented reality (AR) in the medical field, in general, has allowed for the clear previsualization of surgical procedures, has simplified the planning of the interventions themselves, and has increased patients’ comfort [1,2].

AR can be defined as the enhancement of sensory perception by adding additional pieces of information that cannot be perceived through the five senses [3]. It is indeed feasible to augment the amount of information obtained through the conventional physical examination, for instance, the precise location of anatomical structures. Concurrently, it is possible to eliminate superfluous data, thereby streamlining the operating field view and, consequently, the therapeutic treatments. This technology should be distinguished from virtual Reality (VR), which immerses the user in a computer-generated environment devoid of any tangible elements [3,4].

The digital planning of different interventions is commonly performed in dentistry and maxillofacial surgery on bidimensional screens. The lack of tridimensionality severs the experience since it lacks real-time feedback, resulting, oftentimes, in continuous glances at a screen far from the operative area that may distract the operator and induce errors due to lack of coordination [5,6].

Furthermore, a bidimensional image causes the superimposition of spatial information, making the position of overlapping anatomical structures not always distinguishable [7,8,9].

This limitation has been overcome by the introduction of head-mounted displays (HMDs), headsets that are positioned right in front of the operator’s eyes [10].

AR HMDs may work in two different settings: optical see-through (OST) or video see-through (VST).

OST technology allows the user to have a direct view of reality and projected additional data in their line of sight; in VST mode, the virtual content is superimposed over a digital recording of the real world. Real-time cameras are usually mounted on the headset frame, granting a user-like line of sight.

AR/VR technologies with the support of HMD have yielded diverse benefits in the medical field. These include surgery previsualization, which has the potential to enhance clinical outcomes by streamlining the procedure, and for training purposes [11,12,13,14,15,16]. Moreover, clinicians can inspect data acquired from 3D examinations of the patient’s face (3D facial scan), oral cavity and teeth (intraoral scan), and skeletal structures (CBCT) [17,18].

Matching patients’ data allows for the creation of a “virtual patient”, useful not only for clinical analysis but also for therapy planning. These technologies involve oral surgery and prosthodontics at first, with planning and digital manufacturing of prosthetic appliances, both on natural teeth or on implants, based on CBCT data. Planning has improved with extraoral and intraoral scanning, collectively referred to as “patient virtualization”, and it is evolving towards augmented reality. Consequently, the use of these techniques in other fields of dentistry (restorative dentistry, endodontics, oral surgery, and tooth preparation) represents a logical extension of their use [19].

The adoption of AR-based techniques is occurring rapidly across all medical specialties. In 2020, AR was used in the procedure for inserting pedicle screws in the vertebral column, reducing operative difficulties and proving to be a safe procedure [20].

Similar results were obtained by Felix et al. in thoracolumbar pedicle screw placement under AR guidance [21]. Zhu et al. utilized an AR-based neuroendoscopic navigation system for intracerebral hematoma localization and remotion, with high accuracy and feasibility [22]. Lecointre et al. described an AR-based robotic assistance system for laparoscopic surgery, performing a real-time multimodal and temporal fusion of laparoscopic images with preoperative medical images in a porcine model system, being a reliable, safe, and accurate system [23].

AR has found applications in orthopedics and traumatology: Guo et al. proposed a preoperative virtual simulation and intraoperative navigation-assisted fixation AR-based system. It was found that the patient group who underwent the AR system-aided surgery had a significantly shorter operation time and lower blood loss than the conventional surgery group [24]. Chen et al. proposed a navigation system with enhanced arthroscopic information for knee surgery, in which virtual arthroscopic images could reproduce the correct structural information with a mean error of 0.32 mm [25]. A systematic review of AR applications in orthopedic surgery found that AR in orthopedic surgery has the potential to be a time-saving, risk-reducing, radiation-reducing, and accuracy-enhancing technique, and that the application of AR technology for intraoperative navigation appears to be well suited to the field of orthopedic surgery [26]. AR has also provided benefits in craniomaxillofacial surgery and skull base surgery by overcoming the challenges of traditional navigation systems, such as hand–eye coordination and depth perception, improving ergonomics and visualization [27,28]. In hepatobiliary and pancreatic surgery, intraoperative information generated from an AR system provided useful navigation assistance [29]. In addition to surgical fields, AR has been applied to the treatment of psychologic disorders, cognitive impairment, and motor rehabilitation [30,31,32,33]. A recent systematic review stated that AR technology has been shown to improve ergonomics and visualization, as well as reducing operation time and blood loss in minimally invasive surgery procedures, even though the examined studies have been limited to experimental developmental approaches, while the number of clinical trials and systematic reviews is low [34].

AR offers significant advantages in dental education by providing an immersive and interactive learning experience. By overlaying digital information onto real-world images, students can visualize complex anatomical structures in detail and simulate surgical procedures in a safe and controlled environment. This approach enhances the understanding of complex concepts, improves visual and kinesthetic memory, and allows students to acquire practical skills more effectively. Additionally, the ability to repeat simulations without risks to patients enables students to progressively refine their clinical skills, better preparing them for real-world practice [35,36,37,38,39,40].

However, it is not clear if AR can improve clinical treatment precision. The aim of this systematic research is to ascertain whether the utilization of AR-based instruments can enhance the precision of dental procedures. Precision is a critical factor in improving treatment outcomes, reducing errors, and minimizing invasive interventions. By focusing on precision, the review aims to assess how AR contributes to the refinement of dental procedures, ultimately improving patient care and optimizing clinical workflows. This review is necessary to compile and analyze existing evidence, assess the precision improvements AR can bring to various dental procedures, and identify gaps where further research is needed.

## 2. Materials and Methods

### 2.1. Search Strategy

The systematic review was carried out on electronic databases, including Ovid MEDLINE, PubMed, and the Web of Science. No searches of other databases, conference proceedings, or gray literature were performed, as the focus was placed on peer-reviewed studies from well-established databases to ensure a high level of evidence quality and consistency. The date parameter of the paper collation was set from January 2018 to June 2023. The following terms and their combinations were searched: (augmented reality) AND ((dentistry) OR (oral surgery) OR (endodontic) OR (prosthodontic) OR (dental restorative) OR (periodontology) OR (orthodontics) OR (orthognatic), to which “Boolean operators” were applied. The keywords were selected to gather and register as much relevant data as possible.

The search string used was (“augmented reality”) AND (“dentistry” OR “oral surgery” OR “endodontic” OR “prosthodontic” OR “dental restorative” OR “periodontology” OR “orthodontics” OR “orthognathic”) AND (date: [1 January 2018 TO 30 June 2023]).

The following focus question was developed, according to the population, intervention, comparison, and outcome (PICO) study design:

“Does the use of AR-based instruments (I) increase precision (O) of dental interventions (P) compared to non-AR techniques (C)?”

### 2.2. Eligibility Criteria

The full texts of all possibly relevant research papers were chosen, considering the following inclusion criteria:

- Studies comparing variation in the precision of interventions carried out with AR instruments and non-AR techniques.

All types of study designs, including clinical trials, observational studies, case reports, and case series, were considered to ensure the inclusion of a broad range of evidence. This comprehensive approach was adopted to capture as much relevant data as possible on the use of AR in dentistry, allowing for a more thorough and representative analysis of its impact on precision across various dental procedures.

The exclusion criteria that were considered were as follows:

- Research that evaluates the effects of AR instruments without comparing with non-AR techniques;
- Reviews and meta-analyses.
- Papers without the full text being available.
- Papers not in English language.

English studies may not be peer-reviewed at the same level, further complicating their inclusion. This decision ensures consistency in the quality of the reviewed literature and maintains the accuracy of the analysis.

### 2.3. Study Selection and Data Extraction

To reduce bias, two researchers from Messina University (F.P. and G.L.G.) independently conducted the literature search. In cases where there were discrepancies in the results, these were first addressed through thorough discussion between the two reviewers. If a consensus could not be reached, a third senior researcher (R.L.G.) was consulted to resolve the issue. This procedure was applied at each critical phase of the review process, including initial screening, assessment of eligibility for final inclusion, data extraction and analysis, and quality assessment. By employing this structured, three-step approach, we ensured that the study selection process remained rigorous, transparent, and free from bias, enhancing the overall reliability of the review. The following variables were defined in this investigation: author and year, intervention, object of experimentation, technique, field of interest, conclusions.

### 2.4. Risk of Bias Assessment

The evaluation of in vitro studies was set up with a methodological index that uses a checklist for in vitro studies on dental materials (CONSORT). This checklist of items has the purpose of evaluating how the study was designed, analyzed, and interpreted, and uses 14 domains [41]. In vivo studies were assessed according to ROBINS-I [42]. ROBINS-I evaluates risk of bias across 7 domains: confounding, selection of participants into the study, classification of interventions, deviations of intended interventions, missing data, measurement of outcomes, and the selection of reported results. For in vivo studies, risk of bias elements were rated as “yes”, “possibly yes”, “no”, “possibly no”, “no information”, or “not applicable”. Non-RCTs were then classified using the ROBINS-I classification as “low”, “moderate”, “serious”, or “critical risk of bias” depending on whether the extent of bias in the domains could have resulted in a significant bias in the outcomes of interest. The CONSORT checklist for in vitro studies and the ROBINS-I tool for in vivo studies were applied point by point to evaluate the risk of bias in each study. Two independent reviewers (F.P. and G.L.G.) systematically completed these checklists by answering each item in accordance with the criteria outlined by the tools. In cases where there were discrepancies in the evaluations, a third senior reviewer (R.L.G.) was consulted to resolve them. This process ensured that each study was rigorously assessed for bias in a consistent and structured manner. The results of these assessments are summarized in the accompanying tables and further discussed in the subsequent sections.

## 3. Results

### 3.1. Study Selection

**Figure 1:**
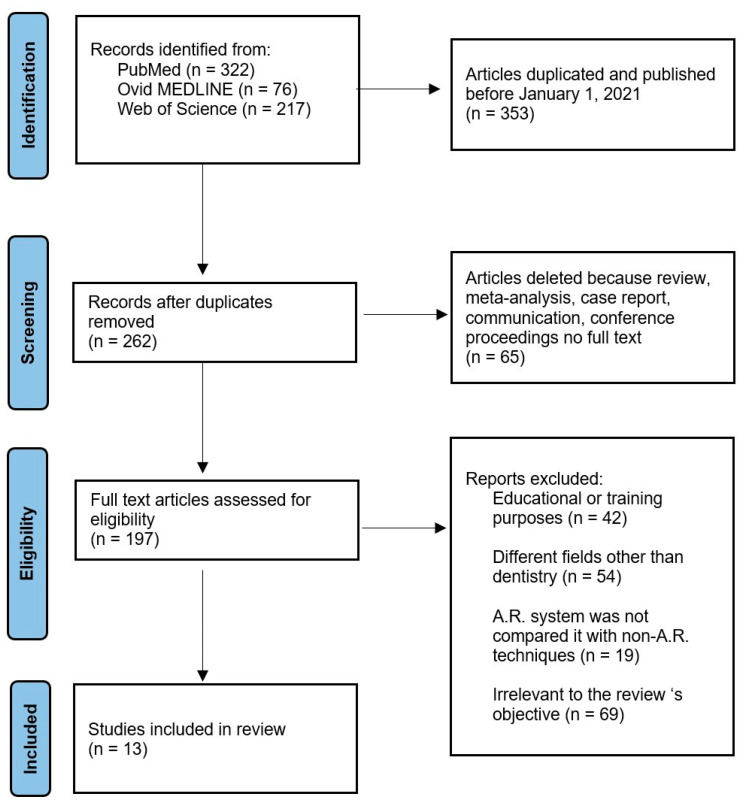
PRISMA flow chart.

The initial search on scientific search engines yielded 615 results. Duplicate research and studies published before 1 January 2021 were excluded, resulting in a total of 262 studies. Out of these, 65 articles were excluded as they were reviews, meta-analyses, case reports, communications, and congress papers. After the initial selection, 197 studies underwent a full-text examination. Among these, 42 articles were discarded because they used AR for educational or training purposes, 54 used AR in fields other than dentistry, 19 used AR but did not compare it with non-AR techniques, and 69 were not aligned with the article. In total, 13 studies were included in this review [43,44,45,46,47,48,49,50,51,52,53,54,55] (Figure 1). The included papers are listed in Table 1.

### 3.2. Risk of Bias

Table 2 and Table 3 present the risk of bias in the in vitro studies and randomized clinical trials (RCTs).

While the results of the risk of bias assessment are presented in the accompanying tables, the primary sources of bias identified across the studies included selection bias, performance bias, and detection bias. Selection bias occurred in studies where the participants were not randomly selected, potentially skewing the results. Performance bias was observed in studies where it was not feasible to blind the participants or clinicians, which may have led to overestimations of the effectiveness of the augmented reality (AR) interventions. Additionally, detection bias was noted in studies that lacked standardized methods for measuring outcomes. These sources of bias may affect the reliability of the results, particularly in evaluating the precision enhancements brought by AR in dentistry. Therefore, caution is needed when interpreting these findings, and future studies should aim to minimize these biases to provide more robust evidence.

In this review, effect measures such as risk ratios or mean differences were not applicable due to the nature and heterogeneity of the included studies, which did not allow for a quantitative synthesis like a meta-analysis. The primary aim was to assess precision improvements in dental procedures using augmented reality, and the diversity of study designs, outcomes, and interventions made direct comparisons challenging. However, where possible, individual study findings related to precision were summarized, and effect sizes were reported qualitatively rather than quantitatively. The narrative synthesis approach was employed due to the diversity of the included study designs, methodologies, and outcome measures. The results of individual studies were combined by identifying common themes, such as the impact of augmented reality on procedural accuracy, patient outcomes, and clinical workflows. Studies were grouped based on their interventions and outcomes, and the key findings were synthesized to highlight patterns and trends in the use of augmented reality across various dental procedures. This approach allowed us to provide a comprehensive overview of the current evidence, despite the heterogeneity of the studies.

Due to the substantial heterogeneity in study designs, outcome measures, and interventions, it was not feasible to conduct a meta-analysis. While individual studies contributed valuable insights into the application of augmented reality (AR) in dentistry, the lack of standardized precision metrics and consistent effect measures made direct comparisons challenging. The included studies varied significantly in their methodology, particularly in how precision was defined and measured, which further complicated any attempt at quantitative synthesis. Therefore, the review adopted a narrative synthesis approach, summarizing and grouping the findings by key themes such as procedural accuracy, patient outcomes, and clinical workflows.

## 4. Discussion

Over the last decade, interest towards medical augmented reality (AR) has increased greatly, fueled by the prospect of developing instruments capable of improving clinical precision [1,2].

This technology could provide significant benefits in accuracy and control during clinical procedures. However, in the dental field, AR uses are still limited.

Nowadays, dental software is developed for implant placement assistance, while other fields of dentistry have not benefited from this technology at the same pace as implant dentistry. At present, there are no commercial AR instruments for assisting in other fields of dentistry (endodontics, restorative dentistry, orthodontics, prosthodontics, periodontal surgery, etc.) but only experimental software. This highlights the need for more specific research to explore and make the most of AR technology’s potential in dentistry.

The research included in this systematic review mainly consisted of in vitro studies [43,44,46,47,48,49,50,51,52,53,54,55]. One in vivo study was also found [45]. Four studies were considered to have a high risk of bias due to the lack of sample randomization, the absence of blinding in the study, and the omission of a power analysis for determining the sample size [44,48,52,54]. These factors significantly compromise the internal validity of the studies, as they increase the likelihood of systematic errors and reduce the potential of obtaining reliable and generalizable results. Nine studies were assessed as having a low risk of bias [43,45,46,47,49,50,51,53,55]. The bias evaluation of the prospective clinical study included in this review gave a result of a “serious risk of bias”. This judgment is due to significant issues identified across various domains, including inadequate control of confounding, potential deviations from the intended intervention, and the risk of selective reporting of effect estimates based on multiple analyses. These factors indicate that the study has important problems that may affect the reliability of its results.

### 4.1. Implant Dentistry

Recently, applications of AR in the dental field have become available for clinical application. These instruments, such as Navident (ClaroNav, Toronto, ON, Canada), X-guide (X-nav technologies, LLC, Lansdale, PA, USA), ImplaNav (ImplaNav, BresMedical, Sydney, Australia), and DENACAM (mininavident AG, Liestal, Switzerland) are used for guided implant surgery. As a natural evolution of static guided implant surgery and thanks to advancements in AR technology, the concept of “dynamic guided implantology” (or “navigated implantology”) has emerged. The planning process follows a similar workflow to that of guided surgery, with an implant project planned based on CBCT imaging, from which a virtual guide is derived [44]. The technique involves first planning the implant procedure using three-dimensional images generated from CBCT. Subsequently, the procedure is carried out using a specific surgical handpiece whose position relative to the patient’s oral cavity is continuously tracked by a system of video cameras, displaying the exact position and angulation of the surgical drill on a screen for the clinician to see [45,46].

Four articles included in this research use AR systems in implant dentistry; one of these uses Navident (ClaroNav, Toronto, ON, Canada) plus a new AR system designed by researchers [50]. The remaining studies (three articles) use systems designed for research purposes [45,54,55].

Kivovics M. et al. and Liu et al. confirm that their AR system designed for implant dentistry allows for better spatial precision (closer real implant position to CBCT implant placement planning) than a freehand technique [46,48]. Other researchers obtained conflicting results [45,50].

González-Rueda JR et al. demonstrated that the conventional freehand technique provides greater accuracy in the placement of zygomatic dental implants than the static computer-assisted implant surgery technique, dynamic computer-assisted implant surgery technique, or augmented reality techniques [50]. In this research, the authors placed the implants in standardized resin blocks, using AR-based techniques, static navigation and dynamic navigation (Navident), and a freehand technique. The results, as explained by the authors, may be justified because the zygomatic dental implants assigned to the freehand control group were the last to be placed, which meant the operator was able to learn and memorize the correct position of the zygomatic dental implants [50]. Santiago Ochandiano et al. evaluated the implant positioning precision in oncologic patients who underwent free flap reconstruction. These patients had their anatomy compromised by previous surgeries for head and neck tumors. The authors used three different techniques for implant placement: static navigation, dynamic navigation, and a combination of static and dynamic navigation; the static–dynamic navigation group obtained the best precision. The observed inaccuracy in the dynamic navigation group is mainly due to the use of a tooth-supported silicone jig. The jig’s instability represented an inaccuracy risk. The authors report that, although the jig allowed for the correct registration, its intraoperative instability led to problems in maintaining the planned position. The jig instability led to involuntary movements during the surgery, making the maintenance of dynamic navigation difficult and causing positioning errors. It must be observed that the patient population consisted of individuals with large anatomical distortions [45].

Placing the implant in the right position significantly impacts the long-term stability and success of implant-supported restoration, particularly in cases where bone availability is limited [56,57].

### 4.2. Endodontics

The advancements in endodontics over recent years can be attributed to the enhancements in NiTi alloys utilized in instruments, which have facilitated minimally invasive access cavities and shaping that more accurately reflects the canal anatomy [58,59,60,61].

No advances have been made that improve the precision in locating the endodontic chamber or the endodontic canals.

Two articles regarding AR-aided endodontic access cavities were included in this systematic review [47,54].

Faus-Matoses et al. positioned extracted single canal teeth in a resin block. They performed the planning of the endodontic access cavity based on a CBCT evaluation, took a scan of the resin block with teeth, and matched the DICOM and STL files. The access cavities were then prepared using the previously planned insertion axis, which is displayed in real time on an HMD (Hololens2, Redmond, WA, USA) [47].

Fangjie Li et al. created an AR system in which root canal therapy is performed visualizing CBCT slices on an HMD, with the aid of a marked high-speed handpiece and mirror, after preoperative planning [54]. Both articles confirm that access cavities performed using dynamic navigation systems are more accurate than those performed using a freehand technique, whether the access cavity is carried out by an expert endodontist or by a non-expert endodontist [47,54]. The use of data obtained from CBCT could be useful to overcome obstacles that may form during the irrigation phases [62].

One study evaluates AR systems for endodontic surgery [44].

Bosshard F.A. et al. created an AR system for endodontic surgery (apicectomy), demonstrating the reliability of the technique compared with static guided surgery [44].

### 4.3. Orthodontics

Three articles included in this systematic review used AR systems to evaluate the precision of orthodontic miniscrew insertion [43,49,52]. After the information obtained from the CBCT images and navigation system was combined on the display device, the AR-aided system indicated the planned miniscrew position to guide the clinicians during the placement of miniscrews, improving the accuracy of miniscrew placement [43,49,52]. In all the included research, the AR-aided systems improved the accuracy of miniscrew placement regardless of the clinician’s level of experience [43,49,52].

### 4.4. Tooth Preparation

Two studies included in this systematic review evaluate the use of AR for tooth preparation [51,55]. The protocol developed by Obispo C. et al. foresees the creation of an acrylic resin 3D printed dental arch with 10 dental elements. Subsequently, ideal tooth preparations for complete crowns were digitally planned using dental planning software according to the tooth preparation guidelines established. Five elements were prepared with a freehand technique, the other five using an AR system that allows for HMD visualization of the virtually planned tooth preparation designs (Hololens1, Redmond, WA, USA) and their superimposition on the resin model [51]. Kihara et al. evaluated the possibility of the implementation of an AR system in order to guide clinicians in tooth preparation, substituting silicone indexes. The system superimposed an ideal abutment shape on a model [55]. The results reported in these articles showed that the computer-aided preparation technique using an augmented reality appliance provided a more accurate preparation design than the freehand preparation technique for complete crown preparation, a more conservative approach with less over-reduction [51,55].

### 4.5. Oral Surgery

A study included in this research evaluated the reliability of an AR system compared to a freehand technique for monoradicular tooth autotransplantation. In this experiment, extracted teeth were mounted on epoxy resin models. The researchers then created “pockets” in the model, allowing transplantation. After digital planning, the teeth were transplanted and subsequently evaluated with a CBCT examination in order to estimate the coronal, apical, and angular deviations. The use of AR allowed for better precision compared to the conventional technique for dental autotransplantations, particularly for apical deviation. These findings suggest that AR could be a promising technique for improving dental autotransplant success [53].

### 4.6. Result Evaluation

The analysis of the studies included in this review demonstrated that the use of AR-based instruments determines a significant precision improvement in dental therapies compared with non-AR-based techniques. The use of AR allows for better accuracy in implant surgery, in endodontic access cavity preparation, and in orthodontic miniscrew placement and tooth preparation, with a more accurate and conservative approach. These findings confirm AR’s potential to revolutionize the therapeutic approach in dentistry, with instruments that enhance accuracy and can reduce intraoperative complications or therapeutic failures.

However, a significant limitation that emerged in this review is the diversity of AR systems used in research. Every study analyzed different software and hardware; this can make it difficult to draw generalizable conclusions. In fact, some instruments may be more or less accurate if compared to similar ones. Plus, the technology that constitutes an instrument may be completely different from another. This lack of standardization underlines the need for unified and efficient AR instrument development, appositely designed for clinical use. Finally, for the majority of AR-based instruments, there needs to be a match between STL data obtained from an intraoral scan and data from a CBCT exam; this determines an increased radiation dose to the patient, even when CBCT scan is not justified, constituting an important limitation of the technique. Recently, an AR-based tool for tracking teeth in a video image for bracket positioning was developed, encompassing this limitation [63]. This innovative approach eliminates the need for CBCT imaging or physical guides, making it a safe and convenient option for clinical use by overlapping the digitally planned bracket position over the patient’s clinical crown for a precise recommendation for bracket positioning. The authors demonstrated the feasibility of the technique but did not measure its accuracy; consequentially, it is not possible to estimate the suitability of this AR-based system [63].

Augmented reality (AR) has not yet been widely applied across all fields of dentistry. For instance, in pediatric dentistry (pedodontics), AR’s potential remains largely untapped. However, recent research has begun exploring its use in this field. A notable example is a study that presented a protocol for developing a serious game aimed at motivating children to practice good oral hygiene habits using AR technology [64]. This innovative approach leverages AR to engage children in an interactive and educational experience, helping them understand the importance of oral hygiene in a fun and immersive way. While the use of AR in fields like pedodontics is still in its early stages, such initiatives highlight its potential for improving patient education and compliance, particularly among younger populations. This also suggests that AR could play a significant role in preventive dentistry, especially in promoting good oral hygiene practices from a young age [64].

Augmented reality (AR) has also been utilized as a non-pharmacological tool to manage chronic or post-operative pain [65]. According to the findings of a systematic review, AR, alongside virtual reality (VR) and mixed reality (MR), has shown promising results in improving pain-related outcomes in various clinical settings. These technologies function by diverting patients’ attention from pain stimuli through immersive experiences, thus promoting pain relief through distraction and cortical re-patterning mechanisms. While most studies observed short-term pain reduction immediately following the intervention, more research is needed to confirm the long-term benefits and address potential accessibility challenges. Moreover, AR-based pain management interventions could also contribute to improving mental health by reducing stress and anxiety associated with chronic pain [65].

Future research might focus on standardized system experiments to allow for a direct comparison between studies, to further validate the AR accuracy in in vitro studies, eliminating the need for CBCT data, limiting ionizing radiation exposure to clinically relevant cases.

### 4.7. Limitations

Most of the studies selected for this research are in vitro studies on models, which limits the clinical applicability of the findings. Therefore, the conclusions of this research may not fully translate into clinical practice. Additionally, the high prevalence of selection, performance, and detection biases across studies may have impacted the reliability of the results. The absence of standardized outcome measures and precision metrics across studies further restricted our ability to conduct a meta-analysis. As a result, our conclusions are primarily based on a qualitative synthesis, which, while informative, does not provide the level of evidence that a quantitative synthesis, such as a meta-analysis, could offer.

Another limitation is time, as AR technology in medicine is a continuously evolving field with rapid advancements in new technologies and devices. The devices or software used in the selected studies may now be outdated or represent outdated versions, potentially explaining some of the discrepancies in the results.

Future research should focus on mitigating these biases by employing more rigorous study designs and establishing standardized outcome measures for precision. This would enable more robust comparisons and allow for a quantitative synthesis to evaluate the overall effectiveness of AR in dental procedures through meta-analysis. Moreover, the field requires well-designed clinical trials to validate the findings observed in vitro and to confirm AR’s benefits in real-world dental practice.

While AR technology presents several potential benefits for enhancing precision and improving outcomes in dental procedures, it also poses certain limitations and risks that must be considered. One of the primary limitations is the high cost associated with the acquisition and implementation of AR systems in dental practices. The technology itself, including the hardware and software, may require significant financial investment, which can be a barrier for smaller clinics or practitioners in developing regions.

From a technical standpoint, AR systems rely on the accuracy of real-time data and the seamless integration of virtual elements with physical environments. Inaccuracies or delays in the AR interface could lead to procedural errors, potentially compromising patient safety. Furthermore, the learning curve for clinicians to effectively use AR systems can be steep, particularly for those who are less familiar with digital tools. This could result in increased procedure times and reliance on the technology, potentially reducing the manual skills of practitioners over time.

In addition to technical and financial concerns, AR also introduces psychological and ergonomic risks for practitioners. The continuous use of AR systems, especially involving wearable devices like headsets or glasses, could contribute to mental fatigue and visual strain, impacting the practitioner’s overall performance. There is also a risk of cognitive overload, where the amount of information presented in real-time through AR systems may overwhelm clinicians, making it difficult to focus on the procedure at hand. In extreme cases, this could contribute to stress and anxiety, potentially affecting mental health [66].

While adverse events related to AR in dentistry are not well documented, early evidence from other medical fields suggests that the extended use of AR tools could lead to a range of ergonomic and mental health issues, including discomfort, disorientation, and burnout. These risks highlight the importance of balancing the use of AR technology with traditional methods, ensuring that clinicians are not overly dependent on these systems and that they receive adequate training and support.

In conclusion, while AR offers promising advancements for dentistry, it is crucial to consider these limitations and risks when implementing the technology in clinical practice. Future research should focus on understanding the long-term effects of AR use on both procedural outcomes and the well-being of healthcare providers.

## 5. Conclusions

The conclusions drawn from this review are consistent with the evidence presented, highlighting the potential of augmented reality (AR) to enhance precision in dental procedures. AR can be applied in clinical practice to improve accuracy in surgical interventions, such as implantology and orthodontics, by providing real-time visual guidance. Additionally, it offers the potential for better training tools in dentistry, allowing practitioners to simulate complex procedures with greater precision. However, several limitations must be acknowledged before AR can be widely integrated into clinical practice. Current AR systems are still in the developmental stages, and the technology’s cost, along with the lack of standardized systems, poses challenges for widespread adoption. Furthermore, many of the studies included in this review are in vitro, limiting the direct application of the findings to real-world clinical settings. To fully realize the potential of AR in dentistry, future research should focus on validating these technologies in clinical trials, developing user-friendly interfaces, and addressing cost and accessibility issues. By overcoming these limitations, AR could become a powerful tool in modern dentistry.

## Figures and Tables

**Table 1:** Data extraction from selected studies.

Author and Year	Intervention	Object	Technique	Field of Interest	Conclusions
Riad Deglow, E., 2021 [43]	Orthodontic miniscrew placement	Resin models using ex vivo material	Computer-aided static navigation (NemoScan®, Nemotec Dental Systems, Madrid, Spain) vs. AR-based technique (Hololens1, Microsoft, Redmond, WA, USA) vs. Conventional technique	Orthodontics	The navigation techniques based on augmented reality technology influence the accuracy of orthodontic self-drilling mini-implant placement and result in fewer intraoperative complications compared to the conventional freehand technique.
Bosshard-Gerber, F., 2022 [44]	Apicectomy	Cadaver pig mandibles	AR-assisted technique (Microsoft HoloLens 2 Augmented Reality Headset, Microsoft, Redmond, WA, USA) vs. Template-guided technique (MED610, Stratasys Ltd., Eden Prairie, MN, USA)	Endodontics	Both methods showed similar accuracy in the ex vivo model.
Ochandiano, S., 2022 [45]	Implant surgery	Oncologic patients treated with implant therapy	New navigation protocol testing	Implant dentistry/oral surgery	The computer-aided implant surgery based on dynamic navigation and 3D-printed surgical modified guides guaranteed accurate implant placement.
Kivovics, M., 2022 [46]	Implant surgery	Three-dimensional printed models	AR-based navigation (Magic Leap One, Plantation, FL, USA) vs. Free technique vs. Static computer-assisted implant surgery (Dental Wings, Dental— Wings Inc., Montreal QC, Canada)	Implant dentistry	The AR-based technique and CAIS showed similar accuracy; both were superior to the freehand technique.
Faus-Matoses, V., 2022 [47]	Endodontic access cavity	Ex vivo specimens	AR-guided technique (Hololens2, Microsoft, Redmond, WA, USA) vs. Freehand technique	Endodontics	The AR technique showed better accuracy compared with the freehand technique.
Liu, L., 2023 [48]	Implant surgery	Resin models	Mixed reality (Hololens, Microsoft, Redmond, WA, USA) vs. Conventional technique	Implant dentistry	The AR-based navigation increased precision in implant surgery compared to the conventional technique, based on entry deviation.
Riad Deglow, E., 2023 [49]	Orthodontic miniscrew placement	Resin models with ex vivo specimens	Two AR-based techniques (Hololens2, Microsoft, Redmond, WA, USA) vs. Conventional technique	Orthodontics	Both AR approaches increased the accuracy in orthodontic miniscrew placement, with less intraoperative complications than freehand technique.
González-Rueda, J.-R., 2023 [50]	Zygomatic implant surgery	Resin models	Static navigation implant surgery vs. Dynamic navigation implant surgery (Navident, ClaroNav, Toronto, ON, Canada) vs. AR-aided implant placement vs. Freehand technique	Implant dentistry	The freehand technique provides greater accuracy of zygomatic dental implant placement than computer-assisted implant surgical techniques, and zygomatic dental implants placed in the anterior region are more accurate than those in the posterior region.
Obispo, C., 2024 [51]	Tooth preparation	Resin models	Freehand technique vs. AR appliance (Hololens1)	Fixed prosthodontics	The AR appliance provides a more conservative and predictable complete crown preparation design than the freehand preparation technique.
Hsu, M.-C., 2024 [52]	Miniscrew placement	Resin models	Conventional technique vs. AR-aided technique	Orthodontics	The AR-aided system improved the accuracy of the miniscrew placement regardless of the clinician’s level of experience.
Marhuenda Ramos, M.T., 2024 [53]	Tooth autotransplantation	Resin models with ex vivo specimens	AR technique (Hololens2) vs. Freehand technique	Oral surgery	The AR appliance provides higher accuracy in the positioning of single-root autotransplanted teeth compared to the conventional freehand technique.
Li, F., 2024 [54]	Root canal treatment	Typodont	New AR-based protocol testing	Endodontics	The protocol ensured better accuracy in simulated root canal treatment.
Kihara, T., 2024 [55]	Tooth preparation	Tooth model	New AR device testing	Fixed prosthodontics	The use of the device allowed for a more conservative approach in tooth preparation.

**Table 2:** In vitro study bias assessment.

Item	Riad Deglow, E., 2021 [43]	Bosshard-Gerber, F., 2022 [44]	Kivovics, M., 2022 [46]	Faus-Matoses, V., 2022 [47]	Liu, L., 2023 [48]	Riad Deglow, E., 2023 [49]	González-Rueda, J.-R., 2023 [50]	Obispo, C., 2024 [51]	Hsu, M.-C., 2024 [52]	Marhuenda Ramos, M.T., 2024 [53]	Li, F., 2024 [54]	Kihara, T., 2024 [55]
1 Abstract	Yes	Yes	Yes	Yes	Yes	Yes	Yes	Yes	Yes	Yes	Yes	Yes
2a Background and objectives	Yes	Yes	Yes	Yes	Yes	Yes	Yes	Yes	Yes	Yes	Yes	Yes
2b Background and objectives	Yes	Yes	Yes	Yes	Yes	Yes	Yes	Yes	Yes	Yes	Yes	Yes
3 Intervention	Yes	Yes	Yes	Yes	Yes	Yes	Yes	Yes	Yes	Yes	Yes	Yes
4 Outcomes	Yes	Yes	Yes	Yes	Yes	Yes	Yes	Yes	Yes	Yes	Yes	Yes
5 Sample size	Yes	No	Yes	Yes	No	Yes	Yes	Yes	No	Yes	No	Yes
6 Randomization: sequence generation	Yes	No	Yes	Yes	No	Yes	Yes	Yes	No	Yes	No	Yes
7 Allocation concealment mechanism	No	No	No	No	No	No	No	No	No	No	No	No
8 Implementation	Yes	No	No	Yes	No	No	No	No	No	No	No	No
9 Blinding	No	No	Yes	No	No	No	No	No	No	No	No	No
10 Statistical methods	Yes	Yes	Yes	Yes	Yes	Yes	Yes	Yes	Yes	Yes	Yes	Yes
11 Results, outcomes, and estimation	Yes	Yes	Yes	Yes	Yes	Yes	Yes	Yes	Yes	Yes	Yes	Yes
12 Discussion and limitations	No	No	Yes	Yes	Yes	Yes	Yes	Yes	Yes	No	No	No
13 Other information and funding	No	Yes	Yes	No	Yes	No	No	No	No	No	No	Yes
14 Protocol	Yes	Yes	Yes	Yes	Yes	Yes	Yes	Yes	Yes	Yes	Yes	Yes

**Table 3:** Prospective clinical study bias assessment. Y: Yes; N: No; PY: Probably Yes; PN: Probably No; NA: Not Applicable.

Bias Domain		Ochandiano, S., 2022 [45]
Bias because of confounding	1.1	Y
	1.2	N
	1.3	NA
	1.4	PN
	1.5	NA
	1.6	N
	1.7	N
	1.8	NA
	RoB judgment	Serious
Bias in selection of participants into the study	2.1	N
	2.2	NA
	2.3	NA
	2.4	Y
	2.5	NA
	RoB Judgment	Low
Bias in classification of interventions	3.1	Y
	3.2	Y
	3.3	NO
	RoB Judgment	Low
Bias because of deviations from intended interventions	4.1	PY
	4.2	PN
	4.3	PY
	4.4	Y
	4.5	PY
	4.6	NA
	RoB Judgment	Low
Bias because of missing data	5.1	Y
	5.2	N
	5.3	N
	5.4	NA
	5.5	NA
	RoB judgment	Low
Bias in measurement of outcomes	6.1	Y
	6.2	Y
	6.3	Y
	6.4	NP
	RoB judgment	Low
Bias in selection of reported results	7.1	PY
	7.2	PY
	7.3	PN
	RoB judgment	Moderate
**Overall bias**		Serious risk of bias
